# A comparison of complementary measures of vitamin B6 status, function, and metabolism in the European Prospective Investigation into Cancer and Nutrition (EPIC) study

**DOI:** 10.1093/ajcn/nqab045

**Published:** 2021-04-07

**Authors:** Joanna L Clasen, Alicia K Heath, Heleen Van Puyvelde, Inge Huybrechts, Jin Young Park, Pietro Ferrari, Mattias Johansson, Ghislaine Scelo, Arve Ulvik, Øivind Midttun, Per Magne Ueland, Christina C Dahm, Jytte Halkjær, Anja Olsen, Theron Johnson, Verena Katzke, Matthias B Schulze, Giovanna Masala, Francesco Segrado, Maria Santucci de Magistris, Carlotta Sacerdote, Marga C Ocké, Leila Luján-Barroso, Ana Ching-López, José María Huerta, Eva Ardanaz, Pilar Amiano, Ulrika Ericson, Jonas Manjer, Björn Gylling, Ingegerd Johansson, Julie Schmidt, Elisabete Weiderpass, Elio Riboli, Amanda J Cross, David C Muller

**Affiliations:** Department of Epidemiology and Biostatistics, School of Public Health, Imperial College London, London, United Kingdom; Department of Epidemiology and Biostatistics, School of Public Health, Imperial College London, London, United Kingdom; International Agency for Research on Cancer, Lyon, France; Department of Public Health and Primary Care, Faculty of Medicine and Health Sciences, Ghent University, Ghent, Belgium; International Agency for Research on Cancer, Lyon, France; International Agency for Research on Cancer, Lyon, France; International Agency for Research on Cancer, Lyon, France; International Agency for Research on Cancer, Lyon, France; Cancer Epidemiology Unit, University of Turin, Turin, Italy; Department of Clinical Science, University of Bergen, Bergen, Hordaland, Norway; Bevital A/S, Bergen, Norway; Department of Clinical Science, University of Bergen, Bergen, Hordaland, Norway; Department of Public Health, Aarhus University, Aarhus, Denmark; Danish Cancer Society Research Center, Diet, Genes and Environment, Copenhagen, Denmark; Danish Cancer Society Research Center, Diet, Genes and Environment, Copenhagen, Denmark; German Cancer research Center (DKFZ), Heidelberg, Germany; German Cancer research Center (DKFZ), Heidelberg, Germany; Department of Molecular Epidemiology, German Institute of Human Nutrition Potsdam-Rehbruecke, Nuthetal, Germany; Institute of Nutritional Science, University of Potsdam, Nuthetal, Germany; Cancer Risk Factors and Life-Style Epidemiology Unit, Institute for Cancer Research, Prevention and Clinical Network - ISPRO, Florence, Italy; Epidemiology and Prevention Unit, Fondazione IRCCS Istituto Nazionale dei Tumori di Milano, Milan, Italy; AOU Federico II, Naples, Italy; Unit of Cancer Epidemiology, Città della Salute e della Scienza University-Hospital, Turin Italy; National Institute for Public Health and the Environment, Bilthoven, The Netherlands; Unit of Nutrition and Cancer, Cancer Epidemiology Research Program, Catalan Institute of Oncology (ICO), L'Hospitalet de Llobregat, Barcelona, Spain; Bellvitge Biomedical Research Institute — IDIBELL, L'Hospitalet de 18 Llobregat, Barcelona, Spain; Escuela Andaluza de Salud Pública (EASP), Granada, Spain; Centro de Investigación Biomédica en Red de Epidemiología y Salud Pública (CIBERESP), Madrid, Spain; Department of Epidemiology, Murcia Regional Health Council, IMIB-Arrixaca, Murcia, Spain; CIBER Epidemiología y Salud Pública (CIBERESP), Madrid, Spain; CIBER Epidemiología y Salud Pública (CIBERESP), Madrid, Spain; Navarra Public Health Institute, Pamplona, Spain; IdiSNA, Navarra Institute for Health Research, Pamplona, Spain; Public Health Division of Gipuzkoa, BioDonostia Research Institute, San Sebastian; CIBER Epidemiología y Salud Pública, Madrid, Spain; Department of Clinical Sciences in Malmö, Lund University, Malmö, Sweden; Dept of Surgery, Skåne University Hospital Malmö, Lund University, Malmö, Sweden; Department of Medical Biosciences, Pathology, Umeå University, Umeå, Sweden; Department of Odontology, Umeå University, Umeå, Sweden; Cancer Epidemiology Unit, Nuffield Department of Population Health, University of Oxford, Oxford, United Kingdom; International Agency for Research on Cancer, Lyon, France; Department of Epidemiology and Biostatistics, School of Public Health, Imperial College London, London, United Kingdom; Department of Epidemiology and Biostatistics, School of Public Health, Imperial College London, London, United Kingdom; Department of Epidemiology and Biostatistics, School of Public Health, Imperial College London, London, United Kingdom; Department of Epidemiology and Biostatistics, School of Public Health, MRC-PHE Centre for Environment and Health, Imperial College London, London, United Kingdom

**Keywords:** vitamin B6, PLP, dietary biomarkers, transsulfuration pathway, kynurenine pathway

## Abstract

**Background:**

Vitamin B6 insufficiency has been linked to increased risk of cancer and other chronic diseases. The circulating concentration of pyridoxal 5′-phosphate (PLP) is a commonly used measure of vitamin B6 status. Ratios of substrates indicating PLP coenzymatic function and metabolism may be useful complementary measures to further explore the role of vitamin B6 in health.

**Objectives:**

We explored the sensitivity of 5 outcomes, namely PLP concentration, homocysteine:cysteine (Hcy:Cys), cystathionine:cysteine (Cysta:Cys), the 3´-hydroxykynurenine ratio (HKr), and the 4-pyridoxic acid ratio (PAr) to vitamin B6 intake as well as personal and lifestyle characteristics.

**Medthods:**

Dietary intake and biomarker data were collected from participants from 3 nested case-control studies within the European Prospective Investigation into Cancer and Nutrition (EPIC). Bayesian regression models assessed the associations of the 5 biomarker outcomes with vitamin B6 intake and personal and lifestyle covariates. Analogous models examined the relations of Hcy:Cys, Cysta:Cys, and HKr with PLP.

**Results:**

In total, 4608 participants were included in the analyses. Vitamin B6 intake was most strongly associated with PLP, moderately associated with Hcy:Cys, Cysta:Cys, and HKr, and not associated with PAr (fold change in marker given a doubling of vitamin B6 intake: PLP 1.60 [95% credible interval (CrI): 1.50, 1.71]; Hcy:Cys 0.87 [95% CrI: 0.84, 0.90]; Cysta:Cys 0.89 [95% CrI: 0.84, 0.94]; HKr 0.88 [95% CrI: 0.85, 0.91]; PAr 1.00 [95% CrI: 0.95, 1.05]). PAr was most sensitive to age, and HKr was least sensitive to BMI and alcohol intake. Sex and menopause status were strongly associated with all 5 markers.

**Conclusions:**

We found that 5 different markers, capturing different aspects of vitamin B6–related biological processes, varied in their associations with vitamin B6 intake and personal and lifestyle predictors.

## Introduction

Vitamin B6 insufficiency has been linked to increased risk for multiple chronic diseases, including cancer, cardiovascular disease, and cognitive decline (1–3). Pyridoxal 5′-phosphate (PLP) is the active form of vitamin B6 and is involved in >160 catalytic functions, including metabolism of amino acids, neurotransmitters, glucose, sphingolipids, and fatty acids (4).

Traditionally, circulating PLP concentration is the most common measure of vitamin B6 status. However, it has been suggested that PLP alone may not capture important biological variation because some metabolic processes requiring PLP are more sensitive to vitamin B6 insufficiency than others (4). It is likely that circulating PLP concentration is influenced by other factors, including age, dietary choices, and medication and substance use, which all influence circulating PLP concentration in a manner that is independent of downstream functions of vitamin B6 (4). For example, variations in PLP concentrations between smokers and nonsmokers may be explained in part by differences in diet and overall nutrient intake (5).

Ratios of substrates to products in PLP-dependent reactions, labeled here as PLP functional markers, can capture aspects of vitamin B6–related enzymatic function (6). These markers are not necessarily expected to correlate closely with PLP concentration because metabolic control is tightly regulated to maintain homeostasis in PLP-dependent reactions, and PLP concentration is often not tightly coupled with availability of products or substrates (7, 8). While functional markers have been previously investigated as potential markers of vitamin B6 status, here we are instead interested in these markers as a representation of a more downstream stage of the metabolic role of PLP compared with the circulating concentration. The proposed functional markers include the ratios homocysteine:cysteine (Hcy:Cys), cystathionine:cysteine (Cysta:Cys), and the HK ratio (HKr), 3´-hydroxykynurenine (HK):[kynurenic acid (KA) + xanthurenic acid (XA) + anthranilic acid (AA) + 3´-hydroxyanthranilic acid (HAA)], the first 2 being indicators of transsulfuration pathway regulation, and the third an indicator of tryptophan catabolism regulation (**Supplementary Figure 1**). Increased Hcy:Cys, Cysta:Cys, and HKr are associated with a higher concentration of substrate relative to products and increased enzymatic regulation (6, 9).

In addition to the functional markers, a ratio has also been developed as an indicator of altered vitamin B6 metabolism during inflammation. 4-Pyridoxic acid (PA) is a downstream catabolite of vitamin B6, which is formed in the liver and excreted in the urine (10). The ratio PA:[PLP + pyridoxal (PL)], shortened to PA-ratio (PAr), represents vitamin B6 metabolism in a broad sense, encompassing variability in catabolism of PLP and its unphosphorylated form (PL) as well as tissue uptake of PL. PAr has been established as a reliable predictor of inflammation and is strongly correlated with other systemic markers of inflammatory status such as C-reactive protein (10).

We propose that a deeper understanding of vitamin B6–dependent mechanisms, explored through determinants of 5 complementary markers representing status, function, and metabolism, would be a helpful prerequisite to exploring their role in disease etiology. We aimed to investigate the sensitivity of PLP, Hcy:Cys, Cysta:Cys, HKr, and PAr to vitamin B6 intake as well as personal and lifestyle characteristics. We also explored associations between circulating PLP and the functional markers.

## Participants and Methods

### Study population

The European Prospective Investigation into Cancer and Nutrition (EPIC) is a prospective cohort study to investigate the associations of diet, lifestyle, and environmental factors with incidence of cancer. The study protocol has been described in further detail previously (11). Briefly, middle-aged adults recruited between 1992 and 2000 completed questionnaires on diet, lifestyle, and medical history, had anthropometric measurements recorded, and provided blood samples. All participants provided informed consent and EPIC was approved by the Ethics Committee of the International Agency for Research on Cancer, Lyon, France, as well as the local ethics committees of the study centers. This investigation includes participants from Denmark, France, Germany, Italy, Spain, Sweden, the Netherlands, and the United Kingdom who were included in nested case-control studies for lung, kidney, and upper aerodigestive tract (UADT) cancers. Cases were matched to controls (1:2 for lung, 1:1 for kidney and UADT) by country, sex, month of blood collection, and year of birth. Details of case ascertainment and control matching are described elsewhere (12–14).

### Dietary assessment

Diet was assessed at recruitment using validated country-specific or study center–specific quantitative or semiquantitative diet assessment methods, including questionnaires (15–17). In a representative subset (7%) of the cohort, 24-h dietary recalls were collected, and standardized nutrient intakes for all countries were calculated for the EPIC nutrient database to improve comparability of intake data across countries (18). Previous research has shown no evidence of substantial variation in vitamin B6 intake between countries in the EPIC study (19). Participants reported whether or not they used any vitamin or mineral supplements; however, data were not collected on the type of supplement taken; therefore, use of supplements containing vitamin B6 is not known for this population.

### Laboratory analyses

Blood fractions were divided into aliquots in 0.5-mL straws, which were heat sealed and stored in liquid nitrogen tanks at −196°C, except in Umeå, Sweden, where samples were stored in 1.8-mL plastic tubes in −80°C freezers, and Denmark, where samples were stored in 1-mL tubes between −120°C and −160°C. Blood draws were done on the same day as the dietary assessment for 63% of participants, within 30 d for an additional 28%, and >30 d apart for 8%. Plasma samples were analyzed at the Bevital laboratory. Biomarkers used in this analysis were PLP, PL, PA, total homocysteine (tHcy), total cysteine (tCys), cystathionine, HK, KA, XA, AA, HAA, folate, cobalamin, and riboflavin. GC-MS was used to assess tHcy, tCys, and cystathionine (20, 21). PLP, PL, PA, riboflavin, and the tryptophan catabolites (HK, KA, XA, AA, and HAA) were analyzed with LC-MS/MS (22). Folate and cobalamin were analyzed using microbiological assays (23, 24).

### Statistical analysis

To allow for comparison of estimates across markers, participants with missing data for any outcome or covariate were excluded from all main analyses. The 5 markers of interest in this study include 1 direct marker (PLP), 3 functional markers (Hcy:Cys, Cysta:Cys, and HKr), and a metabolic marker (PAr). Summary statistics for the markers and covariates within the study sample include proportions for categorical variables and geometric mean and IQR for continuous variables. Bivariate relations are presented as the geometric means (IQRs) for the 5 markers across quartiles or categories of covariates. Correlations between vitamin B6 intake and the 5 markers were estimated with the Pearson correlation coefficient.

All biomarker and nutrient intake variables were log base 2 transformed prior to regression analyses. All continuous predictors were centered at a mean of 0 and scaled to an SD of 1. Hierarchical Bayesian regression models were built to estimate adjusted associations between the markers and vitamin B6 intake (continuous). We assigned normal prior distributions for the coefficients of each covariate with a mean of 0 and an SD equal to the SD of the log-transformed outcome variable. All models were adjusted for total energy intake (continuous, kcal/d), case-control status, and nested case-control study (lung, kidney, UADT). Intercepts were allowed to vary by study center. All covariates were selected a priori based on a literature review. Fully adjusted models were then built with further adjustment for age (continuous, years), BMI (continuous, kg/m^2^), smoking status (never, former, current), sex and menopausal status (men, premenopausal women, postmenopausal women), and alcohol consumption (continuous, g/d). The associations from these models are shown as fold change in the outcome on its original scale and associated 95% credible interval (CrI). For functional markers only, the same models were run with PLP replacing vitamin B6 intake, as well as models further adjusted for additional relevant B vitamins (circulating folate, cobalamin, and riboflavin). In these models the exponentiated coefficients represent the expected fold change in the functional markers for a doubling in concentration of PLP. To facilitate comparison of the strengths of association between the markers, we additionally present results with each of the markers standardized to have an SD of 1 in the **Supplementary Materials**.

Potential interactions of vitamin B6 intake with BMI, alcohol intake, sex and menopause status, and smoking status were assessed by individually adding interaction terms to the adjusted model. Model fits for the original models and the interaction models were compared using the expected log predictive density (ELPD) and the SE of the difference in ELPD between different models, estimated using pareto smoothed importance sampling leave-one-out cross-validation (PSIS LOO-CV) (25). The interaction models were used to estimate the variation in the strength of association between vitamin B6 intake with the 5 markers at given values of the covariates. Analogous interaction models were fit and compared to evaluate whether the associations between circulating PLP and the functional markers vary by these individual-level factors.

As a sensitivity analysis, we excluded participants with diabetes and hypertension at baseline because we aimed to explore vitamin B6 metabolism independent of disease status, and diabetes and hypertension may affect kidney function, which is associated with vitamin B6 metabolism (4). Additionally, in separate models we excluded all participants who were incident cancer cases in the nested case-control studies. To investigate potential confounding from supplement use, we checked the associations of the main predictors with the markers while including supplement use (yes/no) in the models. To assess the models’ sensitivities to the choices of prior distributions, we conducted a sensitivity analysis with weaker prior distributions (prior SD of 2 × the SD of the outcome). We also assessed models with waist-to-hip ratio (WHR) replacing BMI in order to check for variation between measures of body composition. Models were also run excluding only participants with missing covariate or outcome data for each specific model (i.e., participants with missing data for the other 4 outcomes were included) to determine robustness to sample size variation.

We conducted a mediation analysis to determine the extent to which circulating PLP concentration may mediate the association of vitamin B6 intake with the functional markers. Estimates and limits of uncertainty were calculated using draws from the posterior distributions of models with the functional marker regressed on the predictor (vitamin B6 intake) and the mediator (PLP), and the mediator regressed on the predictor. The indirect effect is estimated as the mediator coefficient from the former model multiplied by the predictor coefficient from the latter (26). The proportion mediated is the indirect effect divided by the total effect.

All analyses were conducted using R version 4.0.2 (27), and Bayesian regression models were fit using RStan version 2.21.2 via the package brms version 2.13.0 (28).

## Results

### Population characteristics

Of the 6062 participants in the lung, kidney, and UADT case-control studies, 1454 with missing data were excluded (**Supplementary Figure 2**). Characteristics of the 4608 included participants are summarized by sex in **Table 1**. Of the 5 outcomes, PLP had the most variability, followed by PAr then Cysta:Cys, HKr, and Hcy:Cys with the least variation (based on comparison of IQRs). Denmark had the highest average values for PLP, while the United Kingdom had the highest average vitamin B6 intake (**Supplementary Table 1**).

**TABLE 1:** Vitamin B6–related outcomes, predictors, and covariates for men and women in 3 nested case-control cohorts within the EPIC study^1^

	Female (*n* = 1789)	Male (*n* = 2819)	Total (*n* = 4608)
Vitamin B6 intake, mg/d	1.6 (1.3, 2.1)	2.0 (1.6, 2.5)	1.9 (1.5, 2.4)
PLP, nmol/L	37.0 (24.2, 51.8)	39.8 (27.3, 54.4)	38.7 (25.9, 53.4)
Hcy:Cys	0.039 (0.032, 0.045)	0.043 (0.036, 0.049)	0.042 (0.034, 0.048)
Cysta:Cys	0.00068 (0.00049, 0.00089)	0.00074 (0.00053, 0.00097)	0.00071 (0.00051, 0.00094)
HKr	0.35 (0.29, 0.42)	0.31 (0.26, 0.37)	0.33 (0.27, 0.39)
PAr	0.38 (0.29, 0.49)	0.37 (0.27, 0.49)	0.37 (0.28, 0.49)
Energy intake, kcal/d	1802 (1493, 2187)	2317 (1931, 2803)	2102 (1710, 2585)
Age at recruitment, y	56 (51, 63)	57 (52, 62)	56 (52, 62)
BMI	25.4 (22.7, 28.1)	26.6 (24.4, 29.1)	26.1 (23.7, 28.7)
Alcohol intake, g/d	3.5 (1.4, 12.7)	12.7 (5.6, 39.4)	7.9 (2.6, 28.2)
Country			
Denmark	156 (9%)	432 (15%)	588 (13%)
France	104 (6%)	0 (0%)	104 (2%)
Germany	223 (12%)	682 (24%)	905 (20%)
Italy	310 (17%)	391 (14%)	701 (15%)
Spain	124 (7%)	554 (20%)	678 (15%)
Sweden	95 (5%)	87 (3%)	182 (4%)
The Netherlands	433 (24%)	157 (6%)	590 (13%)
United Kingdom	344 (19%)	516 (18%)	860 (19%)
Smoking status			
Never	848 (47%)	634 (22%)	1482 (32%)
Former	445 (25%)	1095 (39%)	1540 (33%)
Current	496 (28%)	1090 (39%)	1586 (34%)
Menopause status			
NA	0 (0%)	2819 (100%)	2819 (61%)
Premenopausal	283 (16%)	0 (0%)	283 (6%)
Postmenopausal	1506 (84%)	0 (0%)	1506 (33%)
Case-control cohort and status			
Kidney, control	200 (11%)	236 (8%)	436 (9%)
Kidney, case	211 (12%)	239 (8%)	450 (10%)
Lung, control	655 (37%)	926 (33%)	1581 (34%)
Lung, case	317 (18%)	464 (16%)	781 (17%)
UADT, control	207 (12%)	487 (17%)	694 (15%)
UADT, case	199 (11%)	467 (17%)	666 (14%)
Vitamin/mineral supplement use			
Missing	119	298	417
No	982 (59%)	1682 (67%)	2664 (64%)
Yes	688 (41%)	839 (33%)	1527 (36%)
Current oral contraceptive use			
Missing	32	2819	2851
No	1726 (98%)	0	1726 (98%)
Yes	31 (2%)	0	31 (2%)

^1^Values are frequencies (%) or geometric means (IQRs). Abbreviations: Cysta:Cys, cystathionine:cysteine; EPIC, European Prospective Investigation into Cancer and Nutrition; Hcy:Cys, homocysteine:cysteine; HKr, 3´-hydroxykynurenine ratio; NA, not applicable; PAr, 4-pyridoxic acid ratio; PLP, pyridoxal 5′-phosphate; UADT upper aerodigestive tract.

### Correlations

Correlations between vitamin B6 intake and the 5 markers showed wide variation in magnitude (**Supplementary Figure 3**). The strongest correlation was between PLP and HKr (*r* = −0.43). The marker most strongly correlated with vitamin B6 intake was the direct marker, PLP (*r* = 0.19), followed by the functional marker HKr (*r* = −0.13). The 2 transsulfuration markers (Hcy:Cys and Cysta:Cys) were moderately correlated with each other (*r* = 0.13).

### Regression models

Linear associations of vitamin B6 intake alone and vitamin B6 intake together with personal and lifestyle covariates mutually adjusted for each other are shown in **Table 2** (and the same associations for outcomes scaled to an SD of 1 are shown in **Supplementary Table 2**, to allow for comparison across outcomes). For reference, a 1.05-fold change (a 5% increase) corresponds to change from the 50th percentile of the distribution to the 54th, 58th, 54th, 57th, and 54th percentiles for PLP, Hcy:Cys, Cysta:Cys, HKr, and PAr, respectively. In both the minimally adjusted and fully adjusted models, vitamin B6 intake was most strongly associated with PLP, moderately associated with Hcy:Cys, Cysta:Cys, and HKr, and not associated with PAr. A 1.60-fold change in PLP corresponds to going from the 50th to 80th percentile of the sample distribution; likewise, a 0.87-fold change in Hcy:Cys corresponds to going from the 50th to 27th percentile; a 0.89-fold change in Cysta:Cys, from the 50th to 40th percentile; and a 0.88-fold change in HKr, from the 50th to 32nd percentile.

**TABLE 2:** Associations of vitamin B6 intake and other predictors with the 5 vitamin B6 markers in 3 nested case-control cohorts within the EPIC study (*n* = 4608)^1^

Predictor	PLP	Hcy:Cys	Cysta:Cys	HKr	PAr
Minimally adjusted^2^					
Vitamin B6 intake (doubling)	1.62 (1.52, 1.73)	0.84 (0.81, 0.87)	0.91 (0.86, 0.97)	0.88 (0.85, 0.91)	1.01 (0.96, 1.06)
Fully adjusted^3^					
Vitamin B6 intake (doubling)	1.60 (1.50, 1.71)	0.87 (0.84, 0.90)	0.89 (0.84, 0.94)	0.88 (0.85, 0.91)	1.00 (0.95, 1.05)
Age (5 y)	0.98 (0.97, 0.99)	0.99 (0.99, 1.00)	1.02 (1.01, 1.03)	1.01 (1.00, 1.02)	1.06 (1.05, 1.07)
BMI (5 kg/m2)	0.93 (0.91 0.95)	0.96 (0.95, 0.97)	1.06 (1.04, 1.08)	1.01 (1.00, 1.02)	1.03 (1.01, 1.04)
Alcohol intake,^4^ (drinks/d)	1.04 (1.03, 1.05)	1.01 (1.01, 1.02)	0.97 (0.96, 0.98)	1.00 (0.99, 1.00)	0.97 (0.97, 0.98)
Premenopausal women (vs. men)	0.80 (0.74, 0.87)	0.85 (0.82, 0.88)	0.87 (0.82, 0.94)	1.18 (1.13, 1.23)	1.10 (1.04, 1.17)
Postmenopausal women (vs. men)	0.99 (0.94, 1.03)	0.88 (0.86, 0.90)	0.91 (0.88, 0.95)	1.13 (1.10, 1.16)	1.01 (0.97, 1.04)
Smoker, former (vs. never)	0.98 (0.94, 1.02)	1.01 (0.99, 1.03)	1.02 (0.99, 1.06)	1.02 (1.00, 1.05)	1.03 (1.00, 1.06)
Smoker, current (vs. never)	0.80 (0.77, 0.84)	1.08 (1.05, 1.10)	0.98 (0.94, 1.01)	1.06 (1.04, 1.08)	1.11 (1.07, 1.15)

1 Values are fold changes (95% credible intervals). Abbreviations: Cysta:Cys, cystathionine:cysteine; EPIC, European Prospective Investigation into Cancer and Nutrition; Hcy:Cys, homocysteine:cysteine; HKr, 3´-hydroxykynurenine ratio; PAr, 4-pyridoxic acid ratio; PLP, pyridoxal 5′-phosphate.

2 Adjusted only for total energy intake, case-control study, and case status; Bayesian regression with random intercepts for centers.

3 Adjusted for total energy intake, case-control study, case status, vitamin B6 intake, age, BMI, alcohol intake, sex and menopause status, and smoking status; Bayesian regression with random intercepts for centers.

4 12 g alcohol per drink.

Of the 5 markers, PAr had the strongest linear association with age, with a 1.06-fold change in PAr for a 5-year increment in age (95% CrI: 1.05, 1.07). HKr was the least sensitive marker to both BMI and alcohol intake. Sex and menopause status were strongly associated with the 5 markers. Postmenopausal women had lower Hcy:Cys and Cysta:Cys and higher HKr than men, with respective fold changes of 0.88 (95% CrI: 0.86, 0.90), 0.91 (95% CrI: 0.88, 0.95), and 1.13 (95% CrI: 1.11, 1.16). Premenopausal women compared with men had lower PLP (0.80; 95% CrI: 0.74, 0.87) and higher PAr (1.10; 95% CrI: 1.04, 1.17). Compared with never smokers, current smokers had lower PLP and higher estimates for Hcy:Cys, HKr, and PAr; former smokers showed the same trends with weaker magnitude of associations.

The linear associations of Hcy:Cys and HKr with PLP differed somewhat from their associations with vitamin B6 intake (**Table 3** and **Supplementary Table 3**). While vitamin B6 intake had similar strengths of association with the 3 functional markers, the association of PLP with Hcy:Cys (fold change for doubling in PLP 0.94; 95% CrI: 0.93, 0.95) and PLP with Cysta:Cys (fold change: 0.95, 95% CrI: 0.93, 0.96) was weaker than that of PLP with HKr (fold change: 0.88; 95% CrI: 0.87, 0.89).

**TABLE 3:** Associations of PLP and other predictors with 3 functional vitamin B6 markers in 3 nested case-control cohorts within the EPIC study (*n* = 4608)^1^

Predictor	Hcy:Cys	Cysta:Cys	HKr
Minimally adjusted^2^			
PLP (doubling)	0.94 (0.94, 0.95)	0.95 (0.93, 0.96)	0.88 (0.87, 0.89)
Fully adjusted^3^			
PLP (doubling)	0.94 (0.93, 0.95)	0.95 (0.93, 0.96)	0.88 (0.87, 0.89)
Age (5 y)	0.99 (0.99, 1.00)	1.02 (1.01, 1.03)	1.00 (1.00, 1.01)
BMI (5 kg/m2)	0.95 (0.95, 0.96)	1.05 (1.03, 1.07)	0.99 (0.98, 1.00)
Alcohol intake,^4^ (drinks/d)	1.01 (1.01, 1.02)	0.98 (0.97, 0.98)	1.00 (1.00, 1.01)
Premenopausal women (vs. men)	0.84 (0.81, 0.87)	0.85 (0.79, 0.91)	1.12 (1.08, 1.16)
Postmenopausal women (vs. men)	0.89 (0.87, 0.91)	0.90 (0.87, 0.94)	1.12 (1.10, 1.15)
Smoker, former (vs. never)	1.01 (0.99, 1.03)	1.02 (0.98, 1.06)	1.02 (1.00, 1.04)
Smoker, current (vs. never)	1.06 (1.03, 1.08)	0.96 (0.92, 1.00)	1.01 (0.99, 1.04)

1 Values are fold changes (95% credible intervals). Abbreviations: Cysta:Cys, cystathionine:cysteine; EPIC, European Prospective Investigation into Cancer and Nutrition; Hcy:Cys, homocysteine:cysteine; HKr, 3´-hydroxykynurenine ratio; PLP, pyridoxal 5′-phosphate.

2 Adjusted only for case-control study and case status; Bayesian regression with random intercepts for centers.

3 Adjusted for case-control study, case status, PLP, age, BMI, alcohol intake, sex and menopause status, and smoking status; Bayesian regression with random intercepts for centers.

4 12 g alcohol per drink.

After adjusting additionally for circulating folate (vitamin B9) and cobalamin (vitamin B12), the association of PLP with Hcy:Cys was notably closer to 1 (fold change: 0.98; 95% CrI: 0.98, 0.99), and a similar attenuation was seen for the association of PLP with Cysta:Cys (fold change: 0.97; 95% CrI: 0.96, 0.99) (**Table 4**). The association of PLP with HKr was unchanged after additional adjustment for circulating riboflavin (vitamin B2).

**TABLE 4:** Associations of PLP and other predictors with the 3 functional vitamin B6 markers, with Hcy:Cys and Cysta:Cys models additionally adjusted for circulating folate and cobalamin, and HKr model additionally adjusted for circulating riboflavin in 3 nested case-control cohorts, within the EPIC study (*n* = 4608)^1^

	Hcy:Cys	Cysta:Cys	HKr
PLP (doubling)	0.98 (0.98, 0.99)	0.97 (0.96, 0.99)	0.88 (0.87, 0.89)
Age (5 y)	1.00 (0.99, 1.00)	1.02 (1.01, 1.03)	1.00 (1.00, 1.01)
BMI (5 kg/m2)	0.95 (0.95, 0.96)	1.05 (1.03, 1.07)	0.99 (0.98, 1.00)
Alcohol intake,^2^ (drinks/d)	1.02 (1.01, 1.02)	0.98 (0.97, 0.98)	1.00 (1.00, 1.01)
Premenopausal women (vs. men)	0.87 (0.84, 0.90)	0.87 (0.81, 0.93)	1.12 (1.08, 1.16)
Postmenopausal women (vs. men)	0.92 (0.91, 0.94)	0.92 (0.89, 0.95)	1.12 (1.10, 1.14)
Smoker, former (vs. never)	1.01 (0.99, 1.03)	1.02 (0.98, 1.06)	1.02 (1.00, 1.04)
Smoker, current (vs. never)	1.04 (1.02, 1.06)	0.95 (0.91, 0.99)	1.02 (0.99, 1.04)
Folate (doubling)	0.86 (0.86, 0.87)	0.91 (0.89, 0.93)	
Cobalamin (doubling)	0.90 (0.89, 0.91)	1.01 (0.98, 1.03)	
Riboflavin (doubling)			1.01 (1.00, 1.01)

1 Adjusted for case-control study, case status, PLP, age, BMI, alcohol intake, sex and menopause status, smoking status, and folate and cobalamin (Hcy:Cys and Cysta:Cys only) and riboflavin (HKr only); Bayesian regression with random intercepts for centers. Abbreviations: Cysta:Cys, cystathionine:cysteine; EPIC, European Prospective Investigation into Cancer and Nutrition; Hcy:Cys, homocysteine:cysteine; HKr, 3´-hydroxykynurenine ratio; PLP, pyridoxal 5′-phosphate.

2 12 g alcohol per drink.

### Mediation analysis

Mediation analysis showed that 31% (95% CrI: 23%, 41%) of the association between vitamin B6 intake and Hcy:Cys was mediated by PLP concentration, and likewise 31% (95% CrI: 18%, 62%) of the association between vitamin B6 intake and Cysta:Cys, whereas PLP mediated 73% (95% CrI: 58%, 93%) of the association between vitamin B6 intake and HKr.

### Interactions

We found no substantial statistical evidence of interaction between vitamin B6 intake and BMI, alcohol intake, sex and menopause status, or smoking status for any of the 5 markers (**Supplementary Figures 4–8**).

**FIGURE 1:**
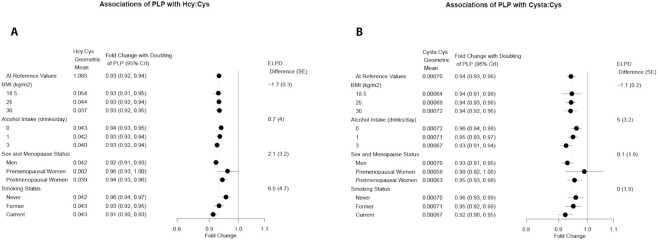
Forest plot of estimated associations of PLP with Hcy:Cys (A) and PLP with Cysta:Cys (B) in 3 nested case-control cohorts within the EPIC study (*n* = 4608). Estimates are at specified covariate levels, holding other predictor variables constant at the mean or reference category. Categories for alcohol are number of drinks per day (12 g alcohol per drink). Values and 95% CrIs are derived from the posterior distributions of Bayesian models with pairwise interaction terms added to the fully adjusted model. The geometric means also assume mean/reference values for other predictors. The ELPD difference and SE of the difference compares the model with interaction term to the original model without interaction. The ELPD difference is positive if the model with an interaction term is a better fit. Abbreviations: CrI, credible interval; Cysta:Cys, cystathionine:cysteine; ELPD, expected log predictive density; EPIC, European Prospective Investigation into Cancer and Nutrition; Hcy:Cys, homocysteine:cysteine; PLP, pyridoxal 5′-phosphate.

**FIGURE 2:**
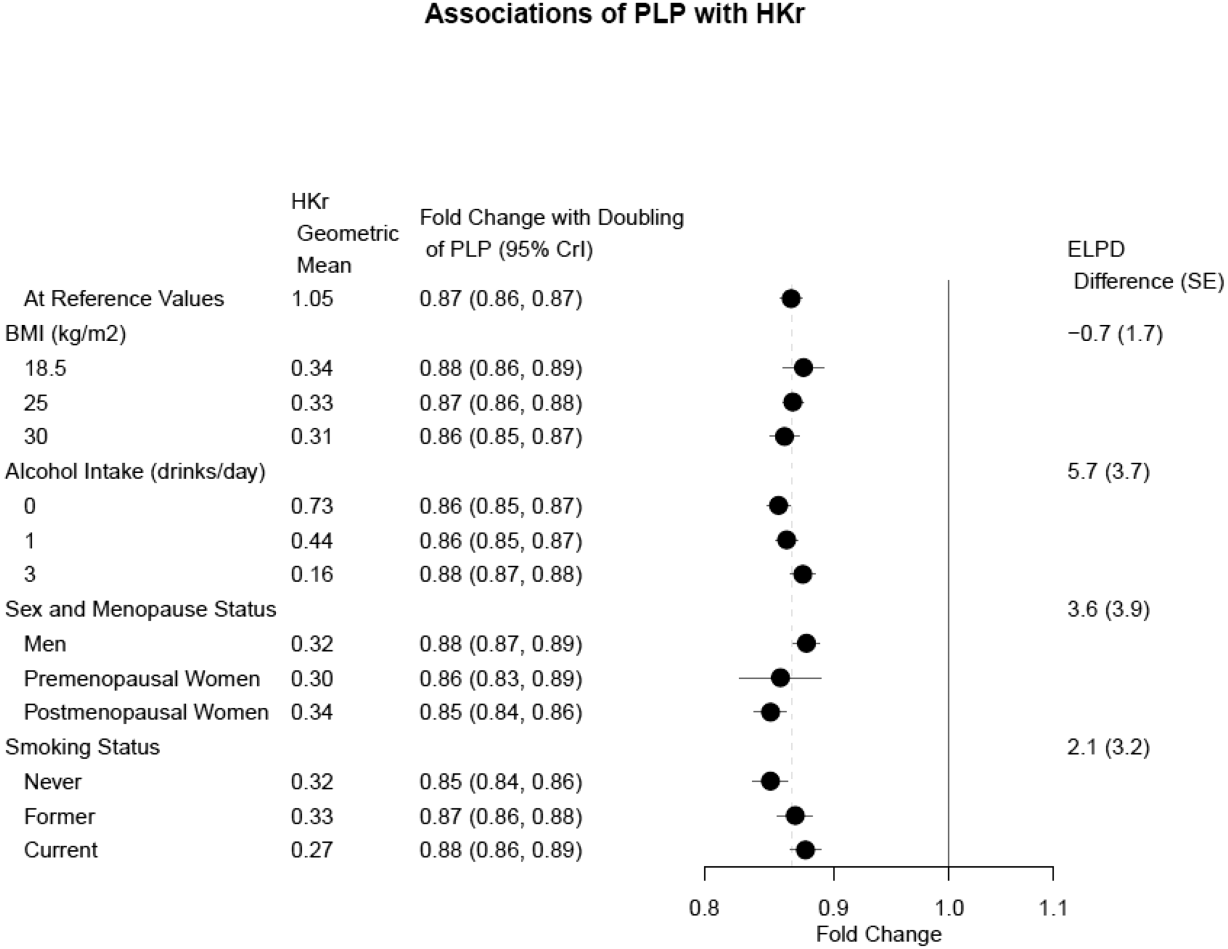
Forest plot of estimated associations of PLP with HKr in 3 nested case-control cohorts within the EPIC study (*n* = 4608). Estimates are at specified covariate levels, holding other predictor variables constant at the mean or reference category. Categories for alcohol are number of drinks per day (12 g alcohol per drink). Values and 95% CrIs are derived from the posterior distributions of Bayesian models with pairwise interaction terms added to the fully adjusted model. The geometric means also assume mean/reference values for other predictors. The ELPD difference and SE of the difference compares the model with interaction term to the original model without interaction. The ELPD difference is positive if the model with an interaction term is a better fit. Abbreviations: CrI, credible interval; ELPD, expected log predictive density; EPIC, European Prospective Investigation into Cancer and Nutrition; HK, 3′-hydroxykynurenine; HKr, HK ratio; PLP, pyridoxal 5′-phosphate.

Similarly, estimates of the associations between PLP and the 3 functional markers did not vary substantially by BMI, and variation by sex and menopause status was accompanied by wide credible intervals (**Figures 1** and **2**). We did find some modest statistical evidence for an interaction with both smoking status and alcohol intake. The association of PLP with Hcy:Cys was stronger among current smokers than never smokers, while the association between PLP and HKr was strongest for never smokers. The association of PLP with HKr also varied by alcohol intake, with nondrinkers having the strongest association. The association of PLP with Cysta:Cys was weakest for nondrinkers.

### Sensitivity analyses

Exclusion of participants with diabetes or hypertension did not notably change the estimates for either vitamin B6 intake or PLP models. Likewise, there was no notable change when excluding the nested case-control cases. The inclusion of supplement use did not materially change any estimates. Using more vague prior distributions did not markedly change the estimates or credible intervals. Associations of WHR with the outcomes were similar to those for BMI. The results of sensitivity analyses for vitamin B6 intake are presented in **Supplementary Table 4**, and for PLP are presented in **Supplementary Table 5**. Estimates had little to no change when the increased sample size (not excluding participants missing data for the other markers) was used (results not shown).

## Discussion

### Principal findings

We evaluated PLP, a direct marker of circulating vitamin B6, Hcy:Cys, Cysta:Cys, and HKr, markers of PLP coenzymatic function, and PAr, a marker of vitamin B6 metabolism. We found that these 5 markers varied in their associations with vitamin B6 intake, with the strongest association for PLP and weakest for PAr. The functional markers varied in their association with PLP, with the strongest association for HKr. Our finding of weaker associations between PLP and the transsulfuration regulation markers after adjustment for folate and vitamin B12 is indicative of strong homeostatic maintenance of an essential metabolic pathway, and suggests that measuring functional as well as direct biomarkers could provide a more complete picture of the role of PLP.

The 5 markers differed in their sensitivity to the individual characteristics age, BMI, alcohol intake, and sex and menopausal status. There were large differences in PLP and PAr between premenopausal women compared with men but not postmenopausal women compared with men. Moreover, large differences in the functional markers between women compared with men were observed, with women having lower Hcy:Cys and Cysta:Cys and higher HKr. Lower Hcy:Cys and Cysta:Cys for women is consistent with suggestive evidence from a mathematical model of one-carbon metabolism that indicated the higher concentration of betaine in women drives the reactions in the transsulfuration pathway (29). The higher HKr among women is also consistent with evidence of kynurenine pathway enzyme inhibition by estrogen (30). Hcy:Cys had inverse associations with BMI and WHR, while Cysta:Cys had positive associations and HKr was not associated with either measure of body composition. There were, however, also some similarities across markers, with estimates for former and current smokers compared with never smokers in the direction of less PLP availability, decreased function (higher Hcy:Cys and HKr), or increased catabolism (higher PAr).

Adjustment for circulating concentrations of other B vitamins involved in the regulation of homocysteine catabolism (folate and cobalamin) attenuated the association between PLP and Hcy:Cys and to a lesser extent the association between PLP and Cysta:Cys. Homocysteine concentration is inversely associated with concentrations of folate and cobalamin, which indirectly activate cystathionine β-synthase, the first PLP-dependent enzyme in the transsulfuration pathway (6, 31). This allosteric regulation has previously been shown to have a greater influence than PLP on homocysteine concentration, and our results of an attenuated PLP-Hcy:Cys association are consistent with this established model of metabolic control (31). Similar attenuation was not seen in the kynurenine pathway—adjustment for circulating concentration of the coenzyme riboflavin did not change the association of PLP with HKr.

We also identified lifestyle characteristics, namely smoking status and alcohol intake, which appeared to influence the strength of association between PLP and the functional markers. While we consider these observations preliminary, this finding—along with the strong associations between the markers and tobacco and alcohol use per se—highlights the possibility that downstream physiological and health effects of tobacco and alcohol use may be in part mediated by pathways dependent on vitamin B6.

Mediation analysis showed that PLP accounted for the majority of the association between vitamin B6 intake and HKr, but a notably smaller proportion of the association between vitamin B6 intake and transsulfuration pathway regulation markers. This indicates that an alternate pathway, not involving PLP, is primarily responsible for the association of vitamin B6 intake with Hcy:Cys and vitamin B6 intake with Cysta:Cys. One possibility is the correlated intake of other B vitamins involved in the broader one-carbon metabolism pathway, including folate and cobalamin (32). Results of these mediation analyses should be interpreted with caution, as the procedure relies on the correct specification of the causal model, which in our case may be undermined by unobserved confounding and simplification/misspecification of the structural model.

### Strengths and limitations

There are limitations that must be considered when interpreting the results of this study. The EPIC cohort only includes participants from Europe, a region in which insufficient vitamin B6 intake is rare (4). Therefore, we were not able to thoroughly evaluate the 5 markers at low levels of vitamin B6 intake or low PLP concentrations, and there is both empirical and theoretical evidence to suggest that the linear trends do not extend to these lower ranges (9). Additionally, this cohort includes primarily middle-aged adults, and therefore a relatively small number of premenopausal women were included, and we were not able to make inferences about the interactions with menopausal status due to a lack of precision in the estimates. A further limitation is the lack of repeat measures over time—while EPIC is a prospective cohort, questionnaires and specimens used in this analysis were collected at a single baseline visit. However, PAr was shown in a longitudinal study to be stable in individuals across time, with an intraclass correlation coefficient of 0.75 for samples drawn 28 d apart (10). The same study also found reasonably high temporal stability for PLP, with an intraclass correlation coefficient of 0.67. Further studies of long-term reproducibility of these markers are needed to understand the utility of single measures in investigation of disease risk.

We were limited to including markers that can be measured in frozen plasma samples, and therefore we could not assess the potential functional markers related to PLP-dependent transaminase activity (4). While there is a long list of potential markers related to vitamin B6 (due to having multiple forms and involvement in a wide range of reactions), the 5 markers included in this analysis represent aspects of vitamin B6 availability, function, and metabolism which are likely to be of interest for analyses of the role of vitamin B6–related pathways and risk of disease.

Self-reported dietary intake assessments may be prone to response bias and food composition tables to measurement errors, which could potentially attenuate the results. We were able to investigate only intake from food because detailed data on supplement use was not available. This limitation is of particular interest for countries with the highest rates of overall supplement use (Denmark and the United Kingdom) and where vitamin B6 is a popular supplement ingredient (the Netherlands) (33); however, sensitivity analyses showed minimal change when adjusting for supplement use. Our analysis of the linear trend of alcohol intake did not allow for exploration of possible variation by alcoholic status, which may be associated with altered homocysteine metabolism (34).

While some factors such as inflammation and kidney function are known to be biologically associated with vitamin B6 status and metabolism (4), there may be a complex feedback loop between them, and this reaches beyond the scope of the current analysis. We have chosen to instead focus on external determinants that are upstream of vitamin B6 metabolism in disease etiology pathways. Additionally, it may be of interest to pursue a more detailed investigation of the transsulfuration regulation markers and their associations with other components of the methionine cycle, including methionine and betaine (6).

A strength of this study was the availability of data for dietary vitamin B6 intake and all 5 markers within a large sample of participants, with all biomarkers measured at the same laboratory. Although this population did not include individuals with very low vitamin B6 intakes, the heterogeneity in this cross-European sample can be considered as a strength. Previous analysis showed variation in PLP between countries in EPIC, with the highest concentrations in Central Europe for men and Northern Europe for women (35). Also, the large battery of subject characteristics available in EPIC is an important strength that allowed appropriate adjustments and sensitivity analyses.

### Related studies

Our findings were consistent with those from a study of the US NHANES population regarding the distributions for PLP by age, sex, smoking status, alcohol intake, and BMI (Pfeiffer et al. reported adjusted estimates of PLP percentage change of −2.1% per 10-y increment in age, −21.2% for women compared with men, −27.6% for yes compared with no smoking, 10.6% for 1 compared with 0 drinks of alcohol per day, and −12.6% for a 25% increment in BMI) (36). Other studies also found directionally consistent associations for PLP with vitamin B6 intake (2, 17, 37–40), BMI (37, 41), cigarette smoking (4, 38), and alcohol intake (4, 38). Predictors of Hcy:Cys were similar to those seen in an analysis of B vitamin status markers in which Hcy:Cys had a modest negative association with BMI and a positive association with smoking and male sex in a Norwegian cohort (6). Predictors of HKr were similar to those from a recent investigation on tryptophan catabolite markers of vitamin B6 status in the same Norwegian cohort, which found a strong negative association of HKr with PLP and a positive association for HKr with smoking (9). As far as we are aware, our analysis is the first comparison of the included 5 vitamin B6 status, function, and metabolism markers with individual-level factors in a single population.

Because of challenges in accurate collection of dietary data and the complexities of metabolic systems, biomarkers of intermediate biological function such as those we have investigated may be useful in clarifying diet–disease associations: not only are the markers less prone to bias in measurement than intake data, but they can also serve as checkpoints within the relevant metabolic systems. Vitamin B6 markers have previously been linked to risk of cancer and other chronic diseases (1), and we anticipate that a deeper understanding of vitamin B6 coenzymatic function can help elucidate the varying roles of different metabolic pathways in disease etiology. Relatively few studies have investigated the functional and catabolic markers in terms of disease risk; however, the emerging evidence suggests an important role for these markers in understanding disease etiology (42–45).

### Summary

We examined 5 complementary markers capturing distinct aspects of vitamin B6–related biological processes in a multinational European study. We found differences in the associations between these markers and vitamin B6 intake, as well as differences in the direction and strength of their associations with personal and lifestyle predictors.

## Supplementary Material

nqab045_Supplemental_File

## Data Availability

For information on how to submit an application for gaining access to EPIC data and/or biospecimens, please follow the instructions at http://epic.iarc.fr/access/index.php.

## Notes

Supported by the Imperial College London President's PhD Scholarship (JLC) and the Cancer Research UK Population Research Fellowship (DCM). The contribution of PF was funded by WCRF (grant 2013/1005). Infrastructure support for the Imperial College London Department of Epidemiology and Biostatistics was provided by the NIHR Imperial Biomedical Research Centre. The coordination of EPIC is financially supported by the European Commission (DG-SANCO) and the International Agency for Research on Cancer. The national cohorts are supported by Danish Cancer Society (Denmark); Ligue Contre le Cancer, Institut Gustave Roussy, Mutuelle Générale de l'Education Nationale, Institut National de la Santé et de la Recherche Médicale (INSERM) (France); German Cancer Aid, German Cancer Research Center (DKFZ), Federal Ministry of Education and Research (BMBF), Deutsche Krebshilfe, Deutsches Krebsforschungszentrum and Federal Ministry of Education and Research (Germany); Associazione Italiana per la Ricerca sul Cancro-AIRC-Italy and National Research Council (Italy); Dutch Ministry of Public Health, Welfare and Sports (VWS), Netherlands Cancer Registry (NKR), LK Research Funds, Dutch Prevention Funds, Dutch ZON (Zorg Onderzoek Nederland), World Cancer Research Fund (WCRF), Statistics Netherlands (The Netherlands); Health Research Fund (FIS-ISCIII), Regional Governments of Andalucía, Asturias, Basque Country, Murcia, Navarra, and the Catalan Institute of Oncology (Barcelona) (Spain); Swedish Cancer Society, Swedish Research Council and County Councils of Skåne and Västerbotten (Sweden); Cancer Research UK (14136 to EPIC-Norfolk; C8221/A19170 and C8221/A29017 to EPIC-Oxford), Medical Research Council (1000143 to EPIC-Norfolk, MR/M012190/1 to EPIC-Oxford) (United Kingdom). Where authors are identified as personnel of the International Agency for Research on Cancer/World Health Organization, the authors alone are responsible for the views expressed in this article and they do not necessarily represent the decisions, policy or views of the International Agency for Research on Cancer/World Health Organization.

Supplemental Tables 1 and 2 and Supplemental Figure 1 are available from the “Supplementary data” link in the online posting of the article and from the same link in the online table of contents at https://academic.oup.com/ajcn/.

Abbreviations used: AA, anthranilic acid; CrI, credible interval; Cysta:Cys, cystathionine:cysteine; ELPD, expected log predictive density; EPIC, European Prospective Investigation into Cancer and Nutrition; HAA, 3′-hydroxyanthranilic acid; Hcy:Cys, homocysteine:cysteine; HK, 3′-hydroxykynurenine; HKr, HK ratio; KA, kynurenic acid; PA, 4-pyridoxic acid; PAr, PA-ratio; PL, pyridoxal; PLP, pyridoxal 5′-phosphate; PSIS LOO-CV, pareto smoothed importance sampling leave-one-out cross-validation; tCys, total cysteine; tHcy, total homocysteine; UADT, upper aero-digestive tract; WHR, waist-to-hip ratio; XA, xanthurenic acid.
