# Supplementary material for: A comparison of complementary measures of vitamin B6 status, function, and metabolism in the European Prospective Investigation into Cancer and Nutrition (EPIC) study
Source: Am J Clin Nutr. 2021 Apr 7;114(1):338–47. doi: 10.1093/ajcn/nqab045 (PMC8246608; doi:10.1093/ajcn/nqab045)
Supplement: nqab045_Supplemental_File [file nqab045_supplemental_file.pdf]

A comparison of complementary measures of vitamin B6 status, function, and metabolism in the European Prospective Investigation into Cancer and Nutrition (EPIC) study.
Clasen et al.

Online Supplementary Material

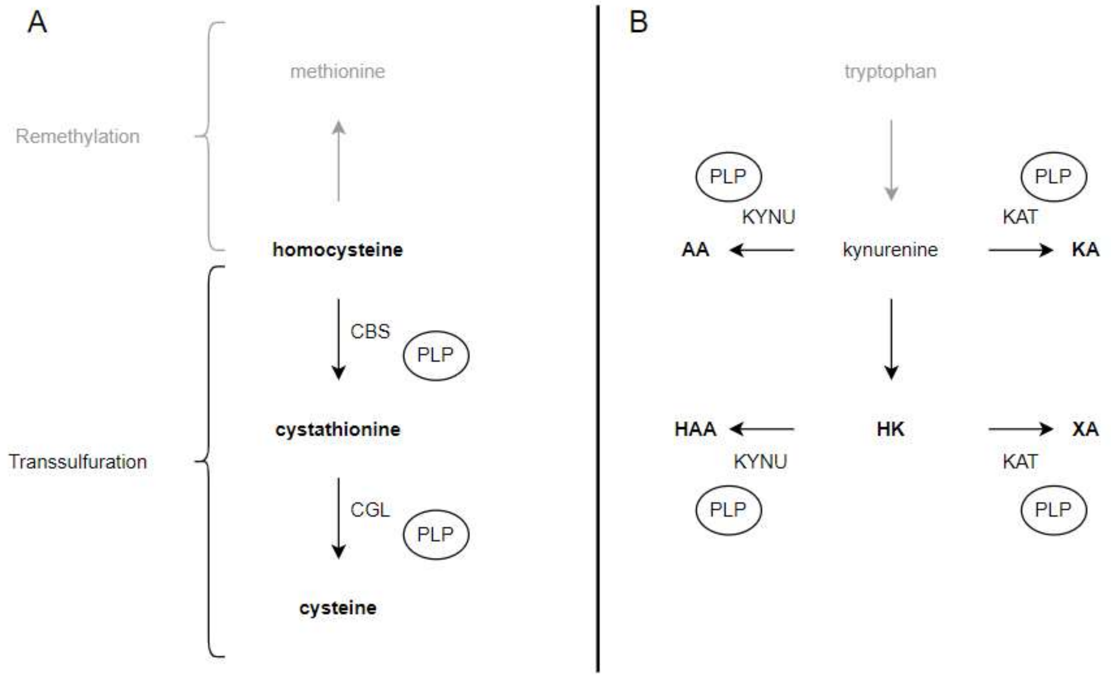

Supplementary Figure 1: Two metabolic pathways that require PLP as a coenzyme: A) Homocysteine catabolism via the transsulfuration pathway, and B) Tryptophan catabolism via the kynurenine pathway. CBS, cystathionine β-synthase; CGL, cystathionine γ-lyase; PLP, pyridoxal 5'-phosphate; KYNU, kynureninase; KAT, kynurenine aminotransferase; HK, 3'-hydroxykynurenine; AA, anthranilic acid; KA, kynurenic acid; HAA, 3'-hydroxyanthranilic acid; XA, xanthurenic acid. Metabolites in bold font are included in the markers of interest (Hcy:Cys, Cysta:Cys, and HKr).

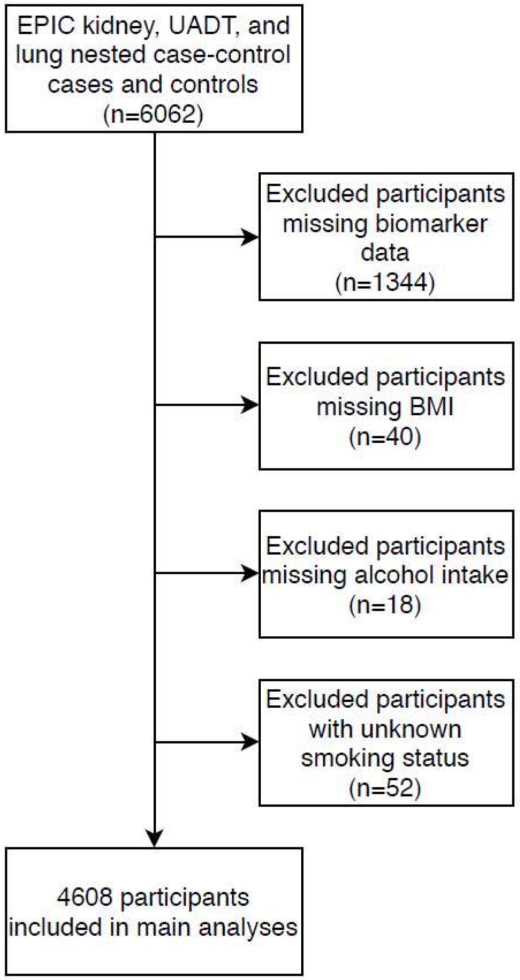

Supplementary Figure 2: Flow chart of participants from three nested case-control cohorts within the European Prospective Investigation into Cancer and Nutrition (EPIC) included in the present analysis.

Supplementary Table 1: Distributions (geometric mean [interquartile range]) of the four markers and vitamin B6 intake by covariate levels or quartiles in three nested case-control cohorts within the EPIC study (n = 4608).

Stratifying Variable	Quartile or Category	PLP	Hcy:Cys	Cysta:Cys	HKr	PAr	Vitamin B6 Intake
Age (years)	34 - <52	37.2 [25.6, 52.1]	0.042 [0.034, 0.049]	0.00065 [0.00048, 0.00084]	0.330 [0.270, 0.397]	0.337 [0.255, 0.443]	1.9 [1.5, 2.4]
Age (years)	52 - <58	39.6 [26.3, 52.9]	0.041 [0.034, 0.047]	0.00073 [0.00052, 0.00094]	0.325 [0.271, 0.388]	0.351 [0.266, 0.458]	1.9 [1.5, 2.4]
Age (years)	58 - <62	39.0 [26.0, 54.4]	0.040 [0.033, 0.046]	0.00071 [0.00050, 0.00095]	0.329 [0.275, 0.394]	0.376 [0.282, 0.494]	1.8 [1.5, 2.2]
Age (years)	62 - 77	38.9 [26.3, 54.2]	0.043 [0.036, 0.050]	0.00077 [0.00058, 0.00100]	0.328 [0.270, 0.396]	0.425 [0.319, 0.561]	1.9 [1.6, 2.5]
BMI (kg/m^2)	14.7 - <23.7	39.1 [25.1, 54.5]	0.044 [0.034, 0.052]	0.00067 [0.00048, 0.00086]	0.338 [0.277, 0.408]	0.374 [0.280, 0.497]	1.8 [1.4, 2.3]
BMI (kg/m^2)	23.7 - <26.1	39.4 [26.4, 55.8]	0.042 [0.035, 0.048]	0.00073 [0.00051, 0.00094]	0.322 [0.268, 0.389]	0.368 [0.278, 0.485]	1.9 [1.5, 2.3]
BMI (kg/m^2)	26.1 - <28.7	39.0 [26.9, 53.3]	0.041 [0.035, 0.047]	0.00070 [0.00051, 0.00092]	0.325 [0.270, 0.387]	0.363 [0.270, 0.474]	1.9 [1.5, 2.4]
BMI (kg/m^2)	28.7 - 65	37.3 [25.8, 51.3]	0.040 [0.033, 0.045]	0.00077 [0.00055, 0.00104]	0.326 [0.269, 0.392]	0.376 [0.281, 0.498]	1.9 [1.5, 2.5]
Alcohol Intake (g/day)	0 - <2	34.2 [23.0, 48.9]	0.041 [0.034, 0.047]	0.00072 [0.00053, 0.00093]	0.345 [0.279, 0.417]	0.405 [0.304, 0.529]	1.7 [1.4, 2.2]
Alcohol Intake (g/day)	2 - <10	38.4 [25.8, 51.6]	0.040 [0.034, 0.046]	0.00073 [0.00052, 0.00098]	0.329 [0.272, 0.394]	0.384 [0.287, 0.497]	1.8 [1.5, 2.3]
Alcohol Intake (g/day)	10 - <27	39.6 [27.2, 54.2]	0.041 [0.034, 0.048]	0.00073 [0.00052, 0.00095]	0.321 [0.269, 0.383]	0.366 [0.276, 0.484]	1.9 [1.5, 2.3]
Alcohol Intake (g/day)	27 - 548	43.0 [29.3, 59.9]	0.044 [0.036, 0.050]	0.00068 [0.00049, 0.00088]	0.317 [0.265, 0.379]	0.331 [0.250, 0.439]	2.1 [1.7, 2.6]
Sex/Menopause Status	Men	39.8 [27.3, 54.4]	0.043 [0.036, 0.049]	0.00074 [0.00053, 0.00097]	0.314 [0.264, 0.373]	0.366 [0.272, 0.487]	2.0 [1.6, 2.5]
Sex/Menopause Status	Premenopausal	32.4 [22.8, 44.0]	0.039 [0.031, 0.045]	0.00060 [0.00044, 0.00073]	0.355 [0.287, 0.427]	0.349 [0.269, 0.437]	1.7 [1.4, 2.1]
Sex/Menopause Status	Postmenopausal	37.9 [24.6, 53.5]	0.039 [0.032, 0.046]	0.00070 [0.00051, 0.00092]	0.350 [0.287, 0.424]	0.382 [0.289, 0.501]	1.6 [1.3, 2.1]
Smoking Status	Never	41.1 [27.8, 56.1]	0.039 [0.033, 0.045]	0.00070 [0.00051, 0.00092]	0.326 [0.272, 0.393]	0.361 [0.273, 0.469]	1.8 [1.5, 2.3]
Smoking Status	Former	41.8 [28.7, 57.7]	0.041 [0.034, 0.048]	0.00075 [0.00053, 0.00096]	0.321 [0.265, 0.385]	0.372 [0.278, 0.488]	1.9 [1.6, 2.4]
Smoking Status	Current	33.9 [22.4, 47.2]	0.044 [0.036, 0.050]	0.00069 [0.00050, 0.00092]	0.337 [0.277, 0.405]	0.378 [0.282, 0.506]	1.8 [1.5, 2.4]
Country	Denmark	44.0 [27.2, 64.6]	0.040 [0.034, 0.044]	0.00090 [0.00060, 0.00122]	0.333 [0.277, 0.392]	0.370 [0.272, 0.499]	2.0 [1.7, 2.5]
Country	France	39.9 [27.3, 52.7]	0.039 [0.034, 0.043]	0.00062 [0.00042, 0.00080]	0.350 [0.299, 0.410]	0.338 [0.273, 0.398]	1.7 [1.4, 2.1]
Country	Germany	42.0 [27.9, 59.9]	0.039 [0.033, 0.044]	0.00073 [0.00051, 0.00100]	0.334 [0.280, 0.391]	0.374 [0.278, 0.504]	1.7 [1.4, 2.1]
Country	Italy	30.7 [22.3, 40.5]	0.040 [0.033, 0.046]	0.00069 [0.00051, 0.00089]	0.339 [0.278, 0.409]	0.342 [0.269, 0.435]	1.6 [1.3, 2.0]
Country	Spain	39.5 [28.8, 53.3]	0.041 [0.033, 0.046]	0.00063 [0.00045, 0.00080]	0.313 [0.262, 0.375]	0.318 [0.246, 0.417]	2.1 [1.8, 2.6]
Country	Sweden	31.5 [20.0, 41.8]	0.035 [0.030, 0.040]	0.00053 [0.00043, 0.00068]	0.309 [0.264, 0.373]	0.448 [0.355, 0.568]	1.5 [1.2, 1.8]
Country	The Netherlands	34.2 [22.6, 46.3]	0.044 [0.037, 0.051]	0.00074 [0.00053, 0.00098]	0.342 [0.276, 0.418]	0.358 [0.276, 0.462]	1.5 [1.3, 1.8]
Country	United Kingdom	43.7 [29.6, 60.3]	0.048 [0.039, 0.056]	0.00072 [0.00056, 0.00092]	0.313 [0.260, 0.380]	0.439 [0.329, 0.576]	2.4 [2.0, 2.8]

[1]Abbreviations: pyridoxal 5’-phosphate (PLP), homocysteine:cysteine (Hcy:Cys), cystathionine:cysteine (Cysta:Cys), 3’-hydroxykynurenine ratio (HKr), 4-pyridoxic acid ratio (PAr), body mass index (BMI)

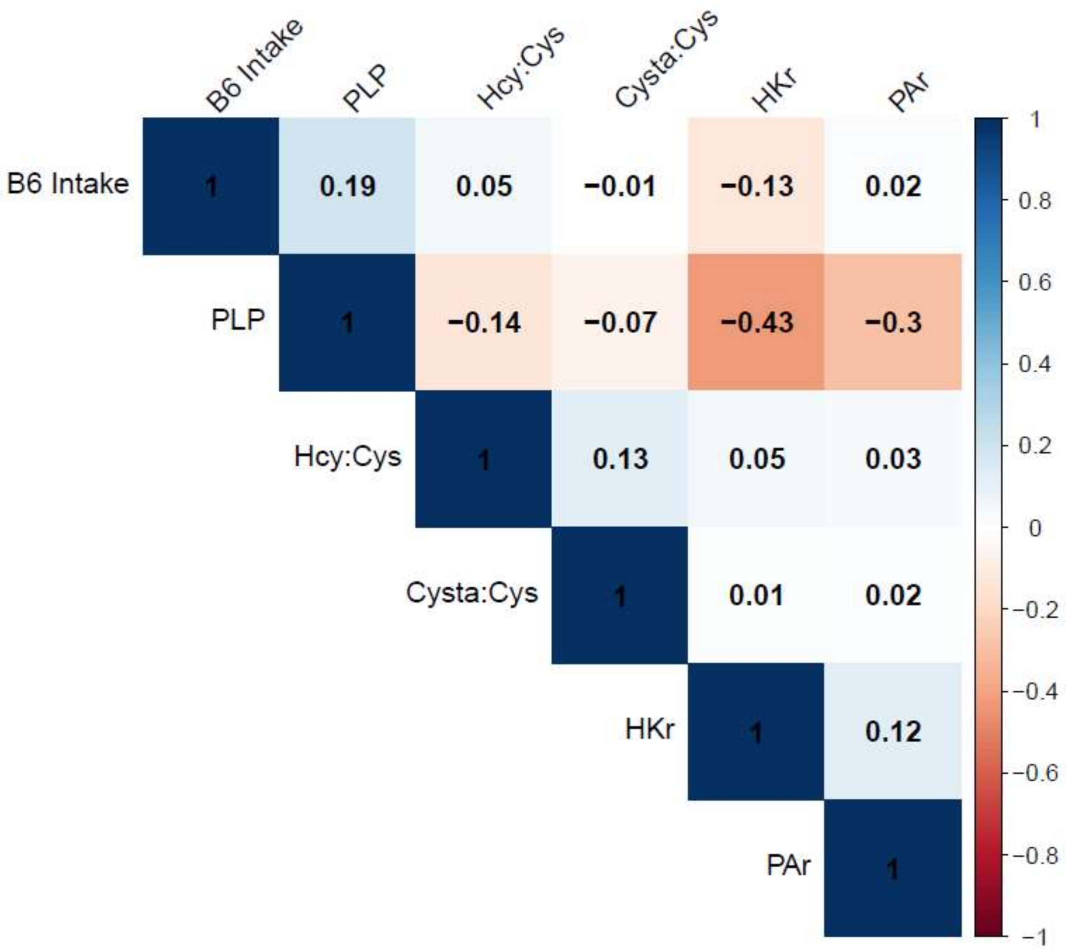

Supplementary Figure 3: Correlations of vitamin B6 intake and the five vitamin B6 markers in three nested case-control cohorts within the EPIC study (n=4608). Abbreviations: pyridoxal 5'-phosphate (PLP), homocysteine:cysteine (Hcy:Cys), cystathionine:cysteine (Cysta:Cys), 3'-hydroxykynurenine ratio (HKr), 4-pyridoxic acid ratio (PAr)

Supplementary Table 2: Associations (fold change [95% credible interval]) of vitamin B6 intake and other predictors with the five vitamin B6 markers scaled to a standard deviation of 1 in three nested case-control cohorts within the EPIC study (n = 4608).

	PLP	Hcy:Cys	Cysta:Cys	HKr	PAr
Vitamin B6 Intake (doubling)	1.70 (1.58, 1.81)	0.73 (0.68, 0.78)	0.85 (0.79, 0.92)	0.74 (0.69, 0.79)	1.00 (0.93, 1.08)
Age (5 years)	0.98 (0.96, 0.99)	0.99 (0.97, 1.00)	1.03 (1.01, 1.05)	1.02 (1.01, 1.04)	1.09 (1.07, 1.11)
BMI (5 kg/m^2)	0.92 (0.90, 0.94)	0.92 (0.90, 0.94)	1.09 (1.06, 1.11)	1.02 (0.99, 1.04)	1.04 (1.02, 1.07)
Alcohol Intake (drinks/day)[1]	1.04 (1.03, 1.05)	1.03 (1.02, 1.04)	0.96 (0.95, 0.97)	0.99 (0.98, 1.00)	0.96 (0.95, 0.97)
Premenopausal Women (vs. Men)	0.78 (0.72, 0.85)	0.69 (0.63, 0.76)	0.83 (0.76, 0.91)	1.47 (1.34, 1.61)	1.16 (1.06, 1.27)
Postmenopausal Women (vs. Men)	0.98 (0.94, 1.03)	0.76 (0.72, 0.79)	0.89 (0.84, 0.94)	1.33 (1.26, 1.40)	1.01 (0.96, 1.06)
Former Smokers (vs. Never)	0.97 (0.93, 1.02)	1.02 (0.98, 1.07)	1.03 (0.98, 1.09)	1.06 (1.01, 1.11)	1.05 (0.99, 1.10)
Current Smokers (vs. Never)	0.78 (0.74, 0.82)	1.18 (1.13, 1.24)	0.97 (0.92, 1.03)	1.15 (1.09, 1.21)	1.18 (1.12, 1.24)

Adjusted for total energy intake, case-control study, case status, and all covariates shown; Bayesian regression with random intercepts for centers

[1] 12g alcohol per drink

Abbreviations: pyridoxal 5'-phosphate (PLP), homocysteine:cysteine (Hcy:Cys), cystathionine:cysteine (Cysta:Cys), 3'-hydroxykynurenine ratio (HKr), 4-pyridoxic acid ratio (PAr), body mass index (BMI)

Supplementary Table 3: Associations (fold change [95% credible interval]) of PLP and other predictors with the three vitamin B6 functional markers scaled to a standard deviation of 1 in three nested case-control cohorts within the EPIC study (n = 4608).

	Hcy:Cys	Cysta:Cys	HKr
PLP (doubling)	0.87 (0.85, 0.89)	0.93 (0.91, 0.95)	0.74 (0.73, 0.76)
Age (5 years)	0.98 (0.97, 1.00)	1.02 (1.01, 1.04)	1.01 (1.00, 1.03)
BMI (5 kg/m^2)	0.90 (0.88, 0.92)	1.07 (1.05, 1.10)	0.98 (0.96, 1.00)
Alcohol Intake (drinks/day)[1]	1.03 (1.02, 1.04)	0.97 (0.96, 0.98)	1.01 (1.00, 1.02)
Premenopausal Women (vs. Men)	0.67 (0.62, 0.73)	0.80 (0.73, 0.88)	1.31 (1.21, 1.43)
Postmenopausal Women (vs. Men)	0.76 (0.73, 0.80)	0.87 (0.83, 0.92)	1.31 (1.25, 1.37)
Former Smokers (vs. Never)	1.02 (0.97, 1.06)	1.03 (0.98, 1.08)	1.05 (1.00, 1.09)
Current Smokers (vs. Never)	1.14 (1.08, 1.19)	0.95 (0.90, 1.00)	1.04 (0.99, 1.09)

Adjusted for case-control study, case status, and all covariates shown; Bayesian regression with random intercepts for centers

[1]12g alcohol per drink

Abbreviations: homocysteine:cysteine (Hcy:Cys), cystathionine:cysteine (Cysta:Cys), 3'-hydroxykynurenine ratio (HKr), pyridoxal 5'-phosphate (PLP), body mass index (BMI)

Associations of Vitamin B6 Intake with PLP

	PLP Geometric Mean	Fold Change with Doubling of Vitamin B6 Intake (95% CrI)	ELPD Difference (SE)
At Reference Values	39.7	1.60 (1.51, 1.71)	
BMI (kg/m2)			−1.4 (1)
18.5	44.6	1.65 (1.51, 1.80)	
25	40.6	1.61 (1.51, 1.72)	
30	37.7	1.58 (1.47, 1.70)	
Alcohol Intake (drinks/day)			10.8 (7.3)
0	37.4	1.68 (1.57, 1.79)	
1	38.8	1.63 (1.53, 1.74)	
3	41.8	1.55 (1.45, 1.65)	
Sex and Menopause Status			0.1 (2.2)
Men	39.7	1.55 (1.45, 1.66)	
Premenopausal Women	31.9	1.69 (1.47, 1.94)	
Postmenopausal Women	39.2	1.67 (1.54, 1.80)	
Smoking Status			−1.9 (1.1)
Never	39.7	1.65 (1.52, 1.78)	
Former	38.7	1.57 (1.45, 1.70)	
Current	31.9	1.60 (1.48, 1.72)	

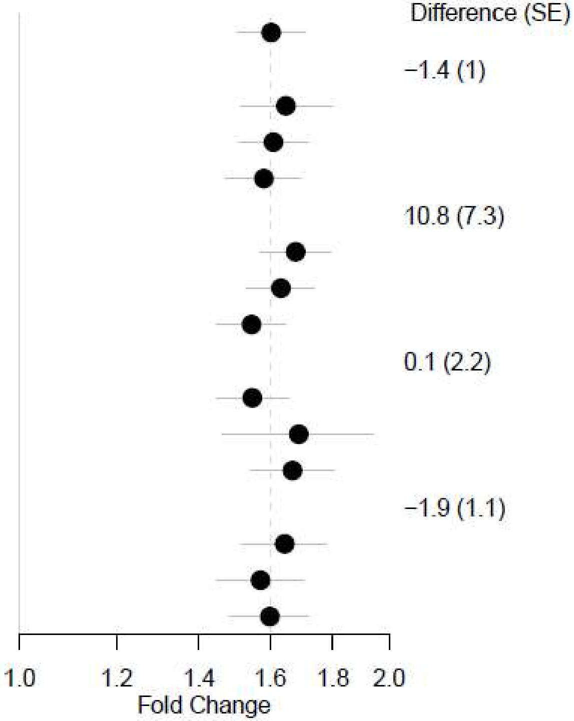

Supplementary Figure 4: Forest plot of estimated associations of vitamin B6 intake with PLP concentration at specified covariate levels, holding other predictor variables constant at the mean or reference category. Values and 95% credible intervals (CrIs) are derived from the posterior distributions of models with pairwise interaction terms added to the adjusted model. The geometric means also assume mean/reference values for other predictors. The expected log predictive density (ELPD) difference and standard error of the difference (SE) compares the model with interaction term to the original model without interaction. The ELPD difference is positive if the model with an interaction term is a better fit. Abbreviations: pyridoxal 5'-phosphate (PLP), body mass index (BMI).

Associations of Vitamin B6 Intake with Hcy:Cys

	Hcy:Cys Geometric Mean	Fold Change with Doubling of Vitamin B6 Intake (95% CrI)	ELPD Difference (SE)
At Reference Values	0.042	0.87 (0.84, 0.90)	
BMI (kg/m2)			−0.4 (0.5)
18.5	0.044	0.87 (0.84, 0.91)	
25	0.042	0.87 (0.84, 0.90)	
30	0.040	0.87 (0.84, 0.90)	
Alcohol Intake (drinks/day)			0.6 (2.4)
0	0.041	0.88 (0.85, 0.90)	
1	0.041	0.87 (0.84, 0.90)	
3	0.042	0.86 (0.84, 0.89)	
Sex and Menopause Status			−0.4 (2.3)
Men	0.042	0.87 (0.84, 0.90)	
Premenopausal Women	0.035	0.82 (0.77, 0.88)	
Postmenopausal Women	0.037	0.88 (0.85, 0.91)	
Smoking Status			−0.3 (1.9)
Never	0.042	0.88 (0.84, 0.91)	
Former	0.042	0.88 (0.85, 0.92)	
Current	0.045	0.85 (0.82, 0.89)	

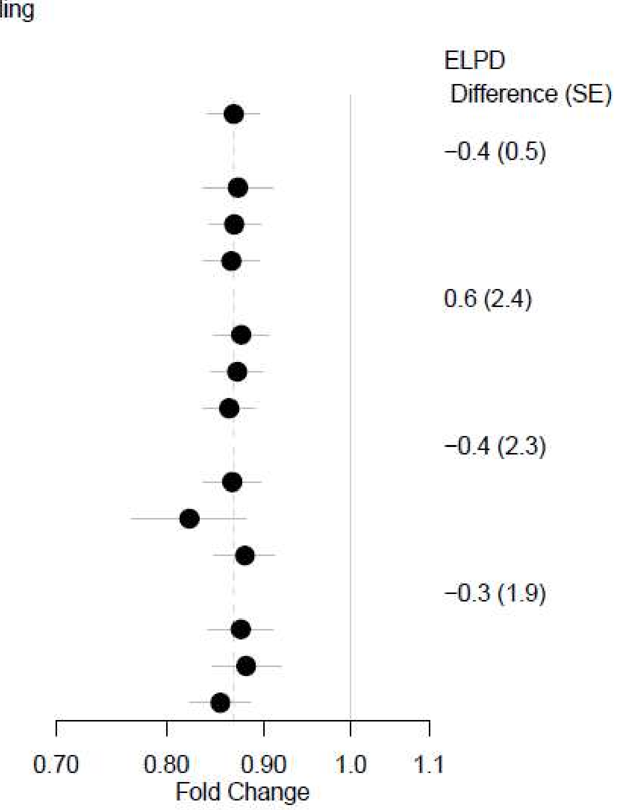

Supplementary Figure 5: Forest plot of estimated associations of vitamin B6 intake with Hcy:Cys at specified covariate levels, holding other predictor variables constant at the mean or reference category. Values and 95% credible intervals (CrIs) are derived from the posterior distributions of models with pairwise interaction terms added to the adjusted model. The geometric means also assume mean/reference values for other predictors. The expected log predictive density (ELPD) difference and standard error of the difference (SE) compares the model with interaction term to the original model without interaction. The ELPD difference is positive if the model with an interaction term is a better fit. Abbreviations: homocysteine:cysteine (Hcy:Cys), body mass index (BMI).

Associations of Vitamin B6 Intake with Cysta:Cys

	Cysta:Cys Geometric Mean	Fold Change with Doubling of Vitamin B6 Intake (95% CrI)	ELPD Difference (SE)
At Reference Values	0.00070	0.89 (0.84, 0.94)	
BMI (kg/m2)			−1.2 (0.2)
18.5	0.00063	0.89 (0.82, 0.96)	
25	0.00068	0.89 (0.84, 0.94)	
30	0.00073	0.89 (0.84, 0.95)	
Alcohol Intake (drinks/day)			0 (1.6)
0	0.00073	0.88 (0.83, 0.93)	
1	0.00071	0.89 (0.84, 0.94)	
3	0.00067	0.90 (0.85, 0.95)	
Sex and Menopause Status			0.6 (2.2)
Men	0.00070	0.92 (0.86, 0.98)	
Premenopausal Women	0.00061	0.82 (0.72, 0.93)	
Postmenopausal Women	0.00064	0.86 (0.81, 0.92)	
Smoking Status			−2.5 (0.6)
Never	0.00070	0.89 (0.83, 0.95)	
Former	0.00071	0.90 (0.84, 0.96)	
Current	0.00068	0.88 (0.83, 0.94)	

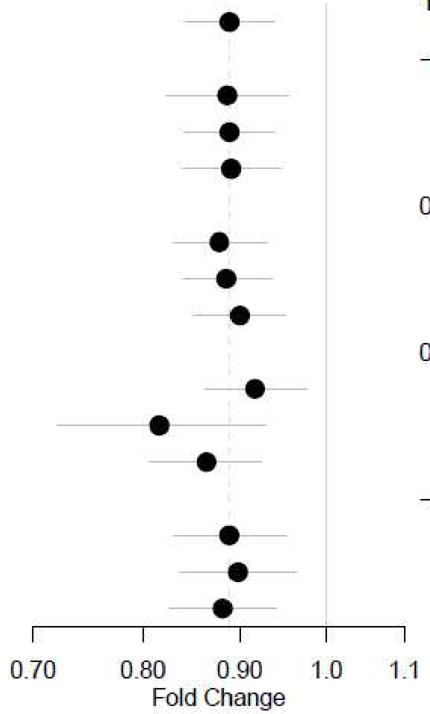

Supplementary Figure 6: Forest plot of estimated associations of vitamin B6 intake with Cysta:Cys at specified covariate levels, holding other predictor variables constant at the mean or reference category. Values and 95% credible intervals (CrIs) are derived from the posterior distributions of models with pairwise interaction terms added to the adjusted model. The geometric means also assume mean/reference values for other predictors. The expected log predictive density (ELPD) difference and standard error of the difference (SE) compares the model with interaction term to the original model without interaction. The ELPD difference is positive if the model with an interaction term is a better fit. Abbreviations: cystathionine:cysteine (Cysta:Cys), body mass index (BMI).

Associations of Vitamin B6 Intake with HKr

	HKr Geometric Mean	Fold Change with Doubling of Vitamin B6 Intake (95% CrI)	ELPD Difference (SE)
At Reference Values	0.32	0.88 (0.85, 0.90)	
BMI (kg/m2)			−0.5 (1.3)
18.5	0.32	0.89 (0.85, 0.93)	
25	0.32	0.88 (0.85, 0.91)	
30	0.32	0.87 (0.84, 0.90)	
Alcohol Intake (drinks/day)			4.6 (3.1)
0	0.32	0.86 (0.83, 0.89)	
1	0.32	0.87 (0.84, 0.90)	
3	0.32	0.89 (0.86, 0.92)	
Sex and Menopause Status			−1.5 (1.5)
Men	0.32	0.89 (0.86, 0.92)	
Premenopausal Women	0.38	0.86 (0.80, 0.92)	
Postmenopausal Women	0.36	0.87 (0.83, 0.90)	
Smoking Status			−1.6 (1.3)
Never	0.32	0.87 (0.84, 0.91)	
Former	0.33	0.87 (0.83, 0.90)	
Current	0.34	0.89 (0.85, 0.92)	

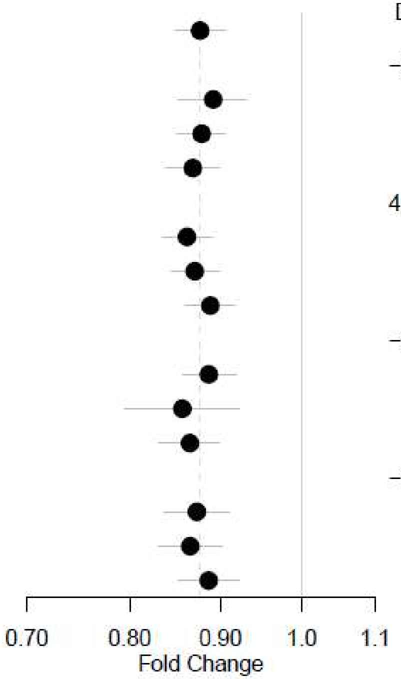

Supplementary Figure 7: Forest plot of estimated associations of vitamin B6 intake with HKr at specified covariate levels, holding other predictor variables constant at the mean or reference category. Values and 95% credible intervals (CrIs) are derived from the posterior distributions of models with pairwise interaction terms added to the adjusted model. The geometric means also assume mean/reference values for other predictors. The expected log predictive density (ELPD) difference and standard error of the difference (SE) compares the model with interaction term to the original model without interaction. The ELPD difference is positive if the model with an interaction term is a better fit. Abbreviations: 3'-hydroxykynurenine ratio (HKr), body mass index (BMI).

Associations of Vitamin B6 Intake with PAr

	PAr Geometric Mean	Fold Change with Doubling of Vitamin B6 Intake (95% CrI)	ELPD Difference (SE)
At Reference Values	0.36	1.00 (0.95, 1.05)	
BMI (kg/m2)			−0.6 (0.8)
18.5	0.34	0.98 (0.92, 1.05)	
25	0.35	1.00 (0.95, 1.05)	
30	0.36	1.01 (0.96, 1.06)	
Alcohol Intake (drinks/day)			9.8 (6.9)
0	0.37	0.97 (0.92, 1.02)	
1	0.36	0.99 (0.94, 1.03)	
3	0.34	1.03 (0.98, 1.08)	
Sex and Menopause Status			−1.3 (1.5)
Men	0.36	1.01 (0.96, 1.07)	
Premenopausal Women	0.39	0.94 (0.84, 1.05)	
Postmenopausal Women	0.36	1.00 (0.94, 1.05)	
Smoking Status			−2 (0.8)
Never	0.36	1.00 (0.94, 1.06)	
Former	0.37	0.99 (0.93, 1.05)	
Current	0.39	1.01 (0.96, 1.07)	

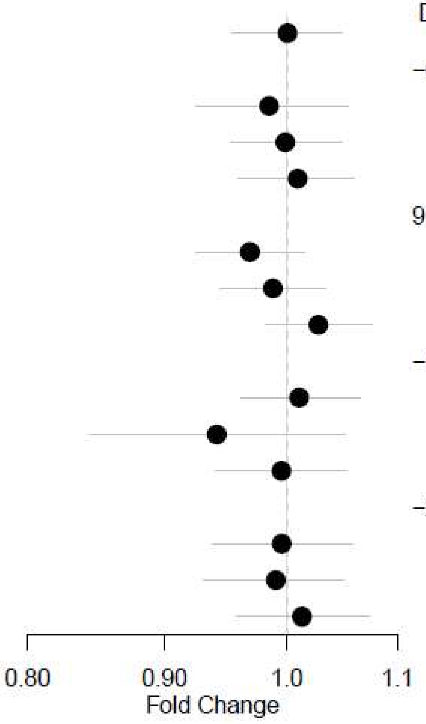

Supplementary Figure 8: Forest plot of estimated associations of vitamin B6 intake with PAr at specified covariate levels, holding other predictor variables constant at the mean or reference category. Values and 95% credible intervals (CrIs) are derived from the posterior distributions of models with pairwise interaction terms added to the adjusted model. The geometric means also assume mean/reference values for other predictors. The expected log predictive density (ELPD) difference and standard error of the difference (SE) compares the model with interaction term to the original model without interaction. The ELPD difference is positive if the model with an interaction term is a better fit. Abbreviations: 4-pyridoxic acid ratio (PAr), body mass index (BMI).

Supplementary Table 4: Sensitivity analyses: Associations (fold change [95% credible interval]) of vitamin B6 intake and other predictors with the five vitamin B6 markers in three nested case-control cohorts within the EPIC study. All models are also adjusted for total energy intake and case-control study, and the last four for case status. Models are hierarchical by center. Healthy Only model excludes participants with diabetes or hypertension.

		PLP	Hcy:Cys	Cysta:Cys	HKr	PAr
Controls Only (n=2711)[1]	Vitamin B6 Intake (doubling)	1.67 (1.54, 1.81)	0.87 (0.84, 0.91)	0.89 (0.83, 0.95)	0.84 (0.81, 0.88)	0.99 (0.93, 1.05)
	Age (5 years)	0.98 (0.96, 0.99)	1.00 (0.99, 1.00)	1.02 (1.01, 1.04)	1.00 (1.00, 1.01)	1.07 (1.05, 1.08)
	BMI (5 kg/m^2)	0.91 (0.89, 0.94)	0.97 (0.96, 0.99)	1.05 (1.03, 1.08)	1.02 (1.00, 1.03)	1.03 (1.01, 1.05)
	Alcohol Intake (drinks/day)[2]	1.05 (1.03, 1.06)	1.01 (1.01, 1.02)	0.97 0.96, 0.98)	0.99 (0.98, 1.00)	0.98 (0.97, 0.99)
	Premenopausal Women (vs. Men)	0.79 (0.72, 0.88)	0.85 (0.81, 0.89)	0.87 (0.79, 0.95)	1.18 (1.12, 1.25)	1.13 (1.05, 1.21)
	Postmenopausal Women (vs. Men)	0.97 (0.92, 1.03)	0.87 (0.85, 0.89)	0.90 (0.86, 0.95)	1.13 (1.10, 1.16)	1.01 (0.97, 1.06)
	Former Smokers (vs. Never)	0.98 (0.93, 1.03)	1.01 (0.99, 1.03)	1.00 (0.96, 1.05)	1.02 (1.00, 1.05)	1.03 (0.99, 1.07)
	Current Smokers (vs. Never)	0.84 (0.79, 0.89)	1.04 (1.01, 1.06)	1.00 (0.95, 1.05)	1.03 (1.00, 1.06)	1.11 (1.07, 1.16)
WHR instead of BMI (n=4394)[3]	Vitamin B6 Intake (doubling)	1.59 (1.50, 1.70)	0.86 (0.83, 0.89)	0.90 (0.85, 0.95)	0.87 (0.85, 0.90)	1.02 (0.97, 1.07)
	Age (5 years)	0.98 (0.97, 1.00)	0.99 (0.99, 1.00)	1.02 (1.01, 1.03)	1.01 (1.00, 1.02)	1.06 (1.04, 1.07)
	WHR	0.54 (0.42, 0.71)	0.77 (0.67, 0.87)	1.76 (1.40, 2.20)	1.07 (0.93, 1.22)	1.32 (1.09, 1.61)
	Alcohol Intake (drinks/day)[2]	1.04 (1.03, 1.05)	1.01 (1.01, 1.02)	0.97 (0.96, 0.98)	1.00 (0.99, 1.00)	0.97 (0.97, 0.98)
	Premenopausal Women (vs. Men)	0.75 (0.69, 0.82)	0.83 (0.80, 0.87)	0.93 (0.86, 1.00)	1.18 (1.13, 1.24)	1.13 (1.06, 1.21)
	Postmenopausal Women (vs. Men)	0.92 (0.87, 0.97)	0.86 (0.83, 0.88)	0.98 (0.93, 1.03)	1.13 (1.10, 1.16)	1.04 (1.00, 1.09)
	Former Smokers (vs. Never)	0.97 (0.93, 1.02)	1.01 (0.99, 1.03)	1.02 (0.98, 1.06)	1.03 (1.00, 1.05)	1.03 (1.00, 1.06)
	Current Smokers (vs. Never)	0.81 (0.78, 0.85)	1.09 (1.06, 1.11)	0.96 (0.92, 1.00)	1.06 (1.03, 1.08)	1.10 (1.06, 1.14)
Healthy Only (n=3337)[3]	Vitamin B6 Intake (doubling)	1.69 (1.57, 1.81)	0.85 (0.82, 0.88)	0.89 (0.84, 0.95)	0.86 (0.83, 0.90)	1.00 (0.95, 1.06)
	Age (5 years)	0.98 (0.96, 0.99)	0.99 (0.98, 1.00)	1.02 (1.00, 1.03)	1.01 (1.00, 1.02)	1.05 (1.04, 1.06)
	BMI (5 kg/m^2)	0.94 (0.92, 0.97)	0.96 (0.95, 0.98)	1.05 (1.02, 1.07)	1.00 (0.99, 1.01)	1.01 (0.99, 1.03)
	Alcohol Intake (drinks/day)[2]	1.04 (1.03, 1.05)	1.01 (1.01, 1.02)	0.97 (0.96, 0.98)	1.00 (0.99, 1.00)	0.97 (0.96, 0.98)
	Premenopausal Women (vs. Men)	0.82 (0.76, 0.90)	0.83 (0.80, 0.87)	0.85 (0.79, 0.91)	1.18 (1.13, 1.23)	1.10 (1.03, 1.17)
	Postmenopausal Women (vs. Men)	1.01 (0.96, 1.07)	0.88 (0.86, 0.90)	0.90 (0.86, 0.94)	1.12 (1.08, 1.14)	1.01 (0.98, 1.05)
	Former Smokers (vs. Never)	0.99 (0.94, 1.04)	1.02 (0.99, 1.04)	1.04 (1.00, 1.09)	1.02 (0.99, 1.05)	1.02 (0.98, 1.05)
	Current Smokers (vs. Never)	0.82 (0.78, 0.86)	1.08 (1.06, 1.11)	0.99 (0.95, 1.04)	1.05 (1.02, 1.08)	1.11 (1.07, 1.16)
Including vitamin/mineral supplement use (n=4191)[3]	Vitamin B6 Intake (doubling)	1.59 (1.49, 1.70)	0.87 (0.84, 0.90)	0.89 (0.84, 0.94)	0.88 (0.85, 0.91)	0.99 (0.94, 1.04)
	Age (5 years)	0.97 (0.96, 0.98)	1.00 (0.99, 1.00)	1.02 (1.01, 1.03)	1.01 (1.00, 1.02)	1.05 (1.04, 1.07)
	BMI (5 kg/m^2)	0.94 (0.92, 0.96)	0.97 (0.95, 0.98)	1.06 (1.04, 1.08)	1.01 (0.99, 1.02)	1.03 (1.01, 1.04)

		PLP	Hcy:Cys	Cysta:Cys	HKr	PAr
	Alcohol Intake (drinks/day)[2]	1.04 (1.03, 1.05)	1.01 (1.01, 1.02)	0.97 (0.96, 0.98)	1.00 (0.99, 1.00)	0.97 (0.96, 0.98)
	Premenopausal Women (vs. Men)	0.79 (0.73, 0.85)	0.85 (0.82, 0.88)	0.87 (0.81, 0.94)	1.19 (1.14, 1.24)	1.07 (1.01, 1.14)
	Postmenopausal Women (vs. Men)	0.97 (0.93, 1.01)	0.89 (0.87, 0.91)	0.92 (0.88, 0.96)	1.14 (1.11, 1.17)	1.00 (0.96, 1.03)
	Former Smokers (vs. Never)	0.97 (0.93, 1.01)	1.01 (0.99, 1.03)	1.03 (0.99, 1.07)	1.03 (1.00, 1.05)	1.02 (0.99, 1.05)
	Current Smokers (vs. Never)	0.80 (0.77, 0.84)	1.08 (1.05, 1.10)	0.99 (0.95, 1.03)	1.06 (1.04, 1.09)	1.11 (1.07, 1.14)
	Vitamin supplement users (vs. non-users)	1.27 (1.22, 1.32)	0.96 (0.94, 0.98)	0.96 (0.93, 1.00)	0.94 (0.92, 0.95)	1.07 (1.04, 1.10)
Prior with Larger SD (n=4608)[3]	Vitamin B6 Intake (doubling)	1.60 (1.51, 1.71)	0.87 (0.84, 0.90)	0.89 (0.84, 0.94)	0.88 (0.85, 0.91)	1.00 (0.96, 1.05)
	Age (5 years)	0.98 (0.97, 0.99)	0.99 (0.99, 1.00)	1.02 (1.01, 1.03)	1.01 (1.00, 1.02)	1.06 (1.05, 1.07)
	BMI (5 kg/m^2)	0.93 (0.91, 0.95)	0.96 (0.95, 0.97)	1.06 (1.04, 1.08)	1.01 (1.00, 1.02)	1.03 (1.01, 1.04)
	Alcohol Intake (drinks/day)[2]	1.04 (1.03, 1.05)	1.01 (1.01, 1.02)	0.97 (0.96, 0.98)	1.00 (0.99, 1.00)	0.97 (0.97, 0.98)
	Premenopausal Women (vs. Men)	0.80 (0.74, 0.87)	0.85 (0.82, 0.88)	0.88 (0.82, 0.94)	1.18 (1.13, 1.23)	1.10 (1.04, 1.17)
	Postmenopausal Women (vs. Men)	0.99 (0.94, 1.03)	0.88 (0.86, 0.90)	0.91 (0.88, 0.95)	1.13 (1.11, 1.16)	1.01 (0.98, 1.04)
	Former Smokers (vs. Never)	0.98 (0.94, 1.02)	1.01 (0.99, 1.03)	1.02 (0.98, 1.06)	1.02 (1.00, 1.05)	1.03 (1.00, 1.06)
	Current Smokers (vs. Never)	0.80 (0.77, 0.84)	1.08 (1.05, 1.10)	0.98 (0.94, 1.01)	1.06 (1.04, 1.08)	1.11 (1.07, 1.15)

[1]Adjusted for total energy intake, case-control study, and all covariates shown; Bayesian regression with random intercepts for centers

[2]12g alcohol per drink

[3]Adjusted for total energy intake, case-control study, case status, and all covariates shown; Bayesian regression with random intercepts for centers

Abbreviations: pyridoxal 5'-phosphate (PLP), homocysteine:cysteine (Hcy:Cys), cystathionine:cysteine (Cysta:Cys), 3'-hydroxykynurenine ratio (HKr), 4-pyridoxic acid ratio (PAr), body mass index (BMI), waist-to-hip ratio (WHR), standard deviation (SD)

Supplementary Table 5: Sensitivity analyses: Associations (fold change [95% credible interval]) of PLP and other predictors with the three functional vitamin B6 markers in three nested case-control cohorts within the EPIC study. All models are also adjusted for case-control study, and the last three for case status. Models are hierarchical by center. The Healthy Only model excludes participants with diabetes or hypertension.

		Hcy:Cys	Cysta:Cys	HKr
Controls Only (n=2711)[1]	PLP (doubling)	0.95 (0.94, 0.96)	0.96 (0.94, 0.98)	0.88 (0.87, 0.89)
	Age (5 years)	0.99 (0.99, 1.00)	1.02 (1.00, 1.04)	1.00 (0.99, 1.01)
	BMI (5 kg/m^2)	0.96 (0.95, 0.98)	1.04 (1.02, 1.07)	0.99 (0.98, 1.01)
	Alcohol Intake (drinks/day)[2]	1.01 (1.01, 1.02)	0.97 (0.96, 0.98)	1.00 (0.99, 1.01)
	Premenopausal Women (vs. Men)	0.84 (0.80, 0.87)	0.84 (0.77, 0.92)	1.12 (1.07, 1.18)
	Postmenopausal Women (vs. Men)	0.88 (0.86, 0.90)	0.89 (0.85, 0.93)	1.12 (1.09, 1.15)
	Former Smokers (vs. Never)	1.01 (0.99, 1.03)	1.00 (0.96, 1.04)	1.02 (0.99, 1.04)
	Current Smokers (vs. Never)	1.03 (1.00, 1.05)	0.99 (0.94, 1.04)	0.99 (0.97, 1.02)
WHR instead of BMI (n=4394)[3]	PLP (doubling)	0.94 (0.93, 0.95)	0.95 (0.93, 0.96)	0.88 (0.87, 0.89)
	Age (5 years)	0.99 (0.99, 1.00)	1.01 (1.00, 1.03)	1.01 (1.00, 1.01)
	WHR	0.72 (0.63, 0.82)	1.65 (1.31, 2.08)	0.94 (0.83, 1.06)
	Alcohol Intake (drinks/day)[2]	1.02 (1.01, 1.02)	0.97 (0.97, 0.98)	1.00 (1.00, 1.01)
	Premenopausal Women (vs. Men)	0.81 (0.78, 0.85)	0.89 (0.83, 0.97)	1.11 (1.07, 1.16)
	Postmenopausal Women (vs. Men)	0.86 (0.83, 0.88)	0.96 (0.91, 1.01)	1.11 (1.08, 1.14)
	Former Smokers (vs. Never)	1.01 (0.99, 1.03)	1.01 (0.98, 1.05)	1.02 (1.00, 1.04)
	Current Smokers (vs. Never)	1.07 (1.05, 1.09)	0.94 (0.90, 0.98)	1.01 (0.99, 1.04)
Healthy Only (n=3337)[3]	PLP (doubling)	0.94 (0.93, 0.95)	0.95 (0.93, 0.97)	0.88 (0.88, 0.89)
	Age (5 years)	0.99 (0.98, 0.99)	1.01 (1.00, 1.03)	1.01 (1.00, 1.01)
	BMI (5 kg/m^2)	0.96 (0.95, 0.97)	1.04 (1.02, 1.06)	0.99 (0.97, 1.00)
	Alcohol Intake (drinks/day)[2]	1.01 (1.01, 1.02)	0.97 (0.97, 0.98)	1.00 (1.00, 1.01)
	Premenopausal Women (vs. Men)	0.82 (0.79, 0.86)	0.83 (0.77, 0.89)	1.13 (1.09, 1.18)

		Hcy:Cys	Cysta:Cys	HKr
	Postmenopausal Women (vs. Men)	0.88 (0.86, 0.90)	0.89 (0.85, 0.93)	1.12 (1.09, 1.14)
	Former Smokers (vs. Never)	1.02 (0.99, 1.04)	1.04 (1.00, 1.08)	1.02 (1.00, 1.04)
	Current Smokers (vs. Never)	1.07 (1.04, 1.10)	0.98 (0.94, 1.02)	1.01 (0.99, 1.04)
Prior with Larger SD (n=4608)[3]	PLP (doubling)	0.94 (0.93, 0.95)	0.95 (0.93, 0.96)	0.88 (0.87, 0.89)
	Age (5 years)	0.99 (0.99, 1.00)	1.02 (1.01, 1.03)	1.00 (1.00, 1.01)
	BMI (5 kg/m^2)	0.95 (0.95, 0.96)	1.05 (1.03, 1.07)	0.99 (0.98, 1.00)
	Alcohol Intake (drinks/day)[2]	1.01 (1.01, 1.02)	0.98 (0.97, 0.98)	1.00 (1.00, 1.01)
	Premenopausal Women (vs. Men)	0.84 (0.81, 0.87)	0.85 (0.79, 0.91)	1.12 (1.08, 1.16)
	Postmenopausal Women (vs. Men)	0.89 (0.87, 0.91)	0.90 (0.87, 0.94)	1.12 (1.10, 1.15)
	Former Smokers (vs. Never)	1.01 (0.99, 1.03)	1.02 (0.98, 1.06)	1.02 (1.00, 1.04)
	Current Smokers (vs. Never)	1.06 (1.04, 1.08)	0.96 (0.92, 1.00)	1.01 (0.99, 1.04)

[1]Adjusted for case-control study and all covariates shown; Bayesian regression with random intercepts for centers

[2]12g alcohol per drink

[3]Adjusted for case-control study, case status, and all covariates shown; Bayesian regression with random intercepts for centers

Abbreviations: pyridoxal 5'-phosphate (PLP), homocysteine:cysteine (Hcy:Cys), cystathionine:cysteine (Cysta:Cys), 3'-hydroxykynurenine ratio (HKr), body mass index (BMI), waist-to-hip ratio (WHR), standard deviation (SD)
